# Hyperspectral Indices Developed from Fractional-Order Derivative Spectra Improved Estimation of Leaf Chlorophyll Fluorescence Parameters

**DOI:** 10.3390/plants13141923

**Published:** 2024-07-12

**Authors:** Jie Zhuang, Quan Wang

**Affiliations:** 1 Graduate School of Science and Technology, Shizuoka University, Shizuoka 422-8529, Japan; zhuang.jie.21@shizuoka.ac.jp; 2 Faculty of Agriculture, Shizuoka University, Shizuoka 422-8529, Japan

**Keywords:** chlorophyll fluorescence, fractional-order derivative, hyperspectral, shaded and sunlit leaves, spectral index

## Abstract

Chlorophyll fluorescence (ChlF) parameters offer valuable insights into quantifying energy transfer and allocation at the photosystem level. However, tracking their variation based on reflectance spectral information remains challenging for large-scale remote sensing applications and ecological modeling. Spectral preprocessing methods, such as fractional-order derivatives (FODs), have been demonstrated to have advantages in highlighting spectral features. In this study, we developed and assessed the ability of novel spectral indices derived from FOD spectra and other spectral transformations to retrieve the ChlF parameters of various species and leaf groups. The results obtained showed that the empirical spectral indices were of low reliability in estimating the ChlF parameters. In contrast, the indices developed from low-order FOD spectra demonstrated a significant improvement in estimation. Furthermore, the incorporation of species specificity enhanced the tracking of the non-photochemical quenching (NPQ) of sunlit leaves (R^2^ = 0.61, r = 0.79, RMSE = 0.15, MAE = 0.13), the fraction of PSII open centers (qL) of shaded leaves (R^2^ = 0.50, r = 0.71, RMSE = 0.09, MAE = 0.08), and the fluorescence quantum yield (ΦF) of shaded leaves (R^2^ = 0.71, r = 0.85, RMSE = 0.002, MAE = 0.001). Our study demonstrates the potential of FOD spectra in capturing variations in ChlF parameters. Nevertheless, given the complexity and sensitivity of ChlF parameters, it is prudent to exercise caution when utilizing spectral indices for tracking them.

## 1. Introduction

Chlorophyll fluorescence (ChlF), which results from the energy level transitions of pigment molecules in photosynthetic antenna complexes after photon capture [1], serves as an indicator of the efficiency and functional integrity of the photosynthetic apparatus [2]. The measurement of fluorescence signals relies on the fluorescence kinetic curve. When dark-adapted plants are exposed to strong actinic light, their fluorescence intensity changes regularly over time, initially increasing, then decreasing, and eventually stabilizing [3]. Using a pulse-amplitude modulation (PAM) fluorescence system, the minimum fluorescence (F_0_) can be measured with weak, modulated light, while the maximum fluorescence (F_m_) is monitored using saturated, pulsed light. Similarly, the minimum and maximum fluorescence under light adaptation can be measured (F’_0_ and F’_m_, respectively). A series of ChlF parameters are derived and calculated, such as maximum photochemical efficiency (PSII_max_), non-photochemical quenching (NPQ), the proportion of open reaction centers in PSII (qL), photochemistry (ΦP), heat dissipation (ΦN), and fluorescence quantum yield (ΦF) [4,5,6,7,8]. These ChlF parameters provide detailed and comprehensive information for evaluating the efficiency of plant photosynthesis and tracking underlying physiological processes [9]. Solar-induced chlorophyll fluorescence (SIF) has gained significant attention for its use of the Fraunhofer line-filling method to invert ChlF, providing new technology and directions for large-scale vegetation fluorescence monitoring [10]. However, SIF signals cannot provide information on parameters such as energy capture, conversion, and dissipation at the photosystem level [9]. Additionally, the decoupling of SIF signals from ecosystem productivity across different spatiotemporal scales, vegetation types, and environmental conditions limits the modeling of fluorescence–photosynthetic coupling models and the estimation of terrestrial ecosystem productivity [11,12,13]. Therefore, robust and practical methods are urgently needed to track ChlF parameters.

Hyperspectral reflectance provides continuous and detailed spectral information which is closely related to plant biochemical and biophysical traits [14]. ChlF parameters are comprehensively determined by these plant traits [15]. Therefore, capturing the variation of ChlF parameters through spectral information is both feasible and efficient. Spectral indices, which combine a limited number of spectral bands, are a convenient method to enhance spectral features sensitive to target parameters [16]. The feasibility of using various spectral indices to track ChlF parameters has been demonstrated [17,18,19]. However, despite some encouraging results, the relationship between spectral indices and ChlF parameters still requires further research and verification. Even the photochemical reflectance index (PRI), which has a clear mechanistic basis, exhibits significant seasonal variation that affects its relationship with NPQ [20]. Similarly, Sonobe and Wang [21] evaluated the relationship between a series of published spectral indices and ChlF parameters. Their study suggested that the relationship between spectral indices and ChlF parameters is influenced by variations in leaf types, species, and stress conditions. Therefore, it is necessary to further validate the effectiveness of spectral indices in tracking ChlF parameters on a multi-species and leaf group (shaded and sunlit leaves) dataset.

Additionally, the relationship between ChlF parameters and leaf biochemical and physical characteristics is complex. Previous studies have shown that leaf mass per area (LMA), chlorophyll content, carotene content, and the ratio of chlorophyll to carotene significantly affect ΦP, ΦF, and qL, while NPQ is sensitive to LMA and equivalent water thickness (EWT) [22]. The response of ChlF parameters to these traits is reflected in their spectral reflectance, influencing the expression of their reflectance. It is imperative to investigate whether spectral features sensitive to ChlF parameters can be parsed using some method and to explore and construct a new type of spectral index. Spectral transformation is recognized for its advantages in noise reduction and accentuating spectral features [23]. A recent study has demonstrated that new spectral indices, constructed based on various spectral transformation methods such as first-order derivatives, logarithms (Log), standard normal variate transformation (SNV), multiplicative scatter correction (MSC), and extended multiplicative scatter correction (EMSC), have improved the predictive performance of ChlF parameters. This highlights the potential of appropriate spectral preprocessing in capturing variations in ChlF parameters [24].

Spectral derivative transformation is considered one of the promising methods in the field of spectral preprocessing. Previous studies typically use integer derivatives to process spectra. For example, the spectral index calculated at 688, 710, and 697 nm of the reflectance spectra based on its first-order derivative has demonstrated good predictive ability for steady-state ChlF [18]. In comparison, the fractional-order derivative (FOD) is an extension of the integer derivative. Compared to the integer derivative, FOD processing can ensure that the signal-to-noise ratio changes slowly and extracts weak features of plants’ spectral reflectance [25]. FOD has shown progress in retrieving leaf traits such as LMA [26], leaf pigment content [27], nitrogen content [28], and photosynthetic capacity parameters [29]. However, estimating ChlF parameters remains an unexplored challenge.

In this study, we focus on developing novel spectral indices derived from FOD spectra and multiple spectral transformations to predict ChlF parameters using a multi-species and leaf group (shaded and sunlit leaves) dataset containing synchronously measured leaf ChlF parameters and spectral reflectance. The objectives of this study are (1) to evaluate the potential of published empirical spectral indices in estimating ChlF parameters; (2) to develop novel spectral indices driven by FOD spectra and multiple spectral transformations for tracking ChlF parameters and to evaluate their performance; and (3) to explore the effects of species specificity and leaf group on the bands of the determined spectral indices.

## 2. Materials and Methods

### 2.1. Measurements and Data Preparation

The experiments were conducted from May to October 2022 and in August 2023 at Nakakawane, a forestry research facility of Shizuoka University, Japan (138°06′ E, 35°04′ N). The experimental materials included *Acer shirasawanum* Koidz, *Betula grossa* Siebold and Zucc., *Carpinus tschonoskii* Maxim, *Fagus crenata* Blume, *Stewartia monadelpha* Siebold and Zucc., and *Pieris japonica* (Thunb.) D. Don ex G. Don. A scaffold tower approximately 15 m high was set up around the experimental site for easy observation and sampling. The detached branch method was strictly adhered to while preparing leaf samples [30]. Branches with target leaf samples were collected from the field before sunrise and promptly transported to the laboratory. All samples were stored in a dark and humid environment before hyperspectral data acquisition and ChlF parameter measurements. This study focused primarily on shaded and sunlit leaves, so leaf samples from the top and bottom of the canopy were screened, and a total of 189 samples were selected.

ChlF measurements were performed using a miniaturized pulse-amplitude-modulated photosynthesis yield analyzer (Mini-PAM, H. Walz, Effeltrich, Germany). Leaf samples that had been dark-adapted for approximately 30 min were measured to obtain F_0_ and F_m_. Subsequently, the leaf samples were subjected to a halogen light source at 600 μmol photons m^−2^ s^−1^ for 20 min to facilitate light adaptation, after which steady-state fluorescence (F_s_) and F’_m_ were measured. The derived ChlF parameters were calculated according to the formulas provided in Table 1.

Leaf reflectance spectra were measured in the spectral range of 350 to 2500 nm, with a 1 nm sampling interval, using a FieldSpec spectrometer (Analytical Spectral Devices, Inc., Boulder, CO, USA). The spectrometer included a leaf clip accessory, which provided a halogen light source and background panels with approximately 100% and 0% reflectance for white and black, respectively. After performing dark current correction and optimization, the reflectance of the white background was recorded as a reference. Leaf reflectance was measured on both white and black background panels, with an average of three replicates. To minimize the impact of machine noise on edge reflectance, the spectra from 400 to 2400 nm were ultimately selected.

### 2.2. Data Processing and Developing New Indices 

The FOD spectra used in this study are based on the Grünwald–Letnikov (G-L) algorithm. The G-L algorithm is widely used in FOD calculations due to its convenience and comprehensiveness [26,29,31]. The G-L algorithm is based on the gamma function, which is as follows [26]:

(1) $$\Gamma \left(\alpha \right)=\int_{0}^{\infty }\exp\left(-U\right)u^{\alpha -1}dU=(\alpha -1)!$$

**Figure 1:**
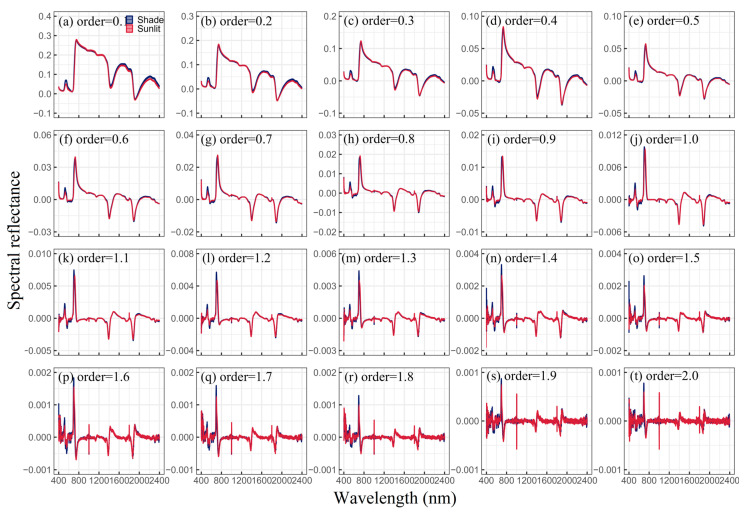
FOD spectra (0.1 to 2, increment of 0.1 per step) for shaded (blue) and sunlit (red) leaves.

**Figure 2:**
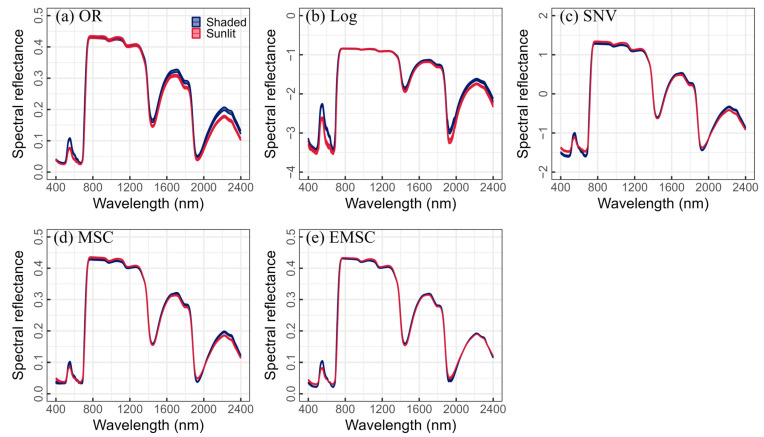
Original and various transformed spectra for shaded (blue) and sunlit (red) leaves.

The FOD spectra at wavelength λ can be calculated using the G-L algorithm as

(2) $$\frac{d^{\alpha }f(\lambda )}{{d\lambda }^{\alpha }}\approx f\left(\lambda \right)+\left(-\alpha \right)f\left(\lambda -1\right)+\frac{\left(-\alpha \right)\left(-\alpha +1\right)}{2}f\left(\lambda -2\right)+\frac{\left(-\alpha \right)\left(-\alpha +1\right)\left(-\alpha +3\right)}{6}f\left(\lambda -3\right)+\ldots +\frac{\Gamma \left(-\alpha +1\right)}{n!\Gamma (-\alpha +n+1)}f\left(\lambda -n\right)$$

where α and *f*(λ) represent the fractional order and reflectance at wavelength λ, respectively, with n = λ/Δλ. The FOD spectra used in this study ranged from 0.1 to 2, with intervals of 0.10 (Figure 1). In addition, Log, SNV, MSC, and EMSC were used as additional spectral transformations (Figure 2).

New spectral indices were derived from the original spectra, FOD, and other spectral transformations. These indices were developed based on different spectral index types. All possible wavelength combinations of a given index were examined at 10 nm intervals [24], and the optimal spectral index was determined based on the AIC criterion. Nine spectral index types were used in this study (Table 2). In addition, ten published empirical spectral indices were selected based on their good performance in previous studies (Table S1) [17,19,21,24]. 

### 2.3. Statistical Analysis

The statistical analysis and visualization were conducted using R version 4.3.0. For each ChlF parameter, the mean, median, minimum, maximum, and coefficient of variation (CV, %) were calculated. Furthermore, the kurtosis and skewness of each parameter were calculated to assess the data’s distribution. A linear regression analysis was utilized to investigate the relationship between the empirical spectral indices and developed spectral indices in conjunction with the ChlF parameters. A multiple linear regression analysis was conducted to establish a model for tracking ChlF parameters, combining the optimal spectral index with species-specific effects. Bootstrapping with 1000 resamplings was employed to train the data. Furthermore, the relative importance of the variables in the model was calculated using the R package ‘relaimpo’ [32]. The coefficient of determination (R), Pearson coefficient (r), root mean square error (RMSE), and mean absolute error (MAE) were employed to assess the efficacy of the model.

## 3. Results

### 3.1. Statistical Descriptions of ChlF Parameters

**Figure 3:**
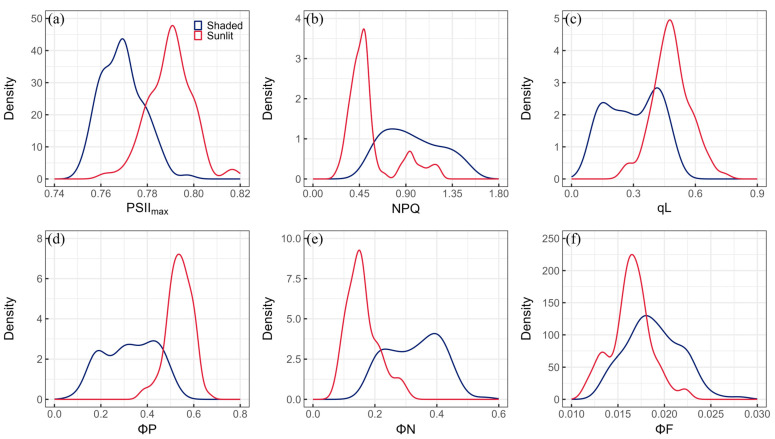
Distribution of (**a**) PSII_max_, (**b**) NPQ, (**c**) qL, (**d**) ΦP, (**e**) ΦN, and (**f**) ΦF for shaded (blue) and sunlit (red) leaves.

The statistical results and the distribution of the ChlF parameters for shaded and sunlit leaves are presented in Table 3 and Figure 3. The mean, median, minimum, and maximum values of the PSII_max_ for shaded leaves were 0.77, 0.77, 0.75, and 0.80, respectively. In contrast, the mean and median values for sunlit leaves were 0.79, with minimum and maximum values of 0.76 and 0.82, respectively. The CV values of the PSII_max_ were low for shaded and sunlit leaves, with values of 1.16% and 1.21%, respectively. The distribution of the PSII_max_ for shaded leaves was primarily concentrated around 0.77, exhibiting slight right skewness (skewness = 0.40) and lighter tails (kurtosis = −0.18). For sunlit leaves, the range of the PSII_max_ that contained the largest proportion was around 0.79, displaying negative skewness (skewness = −0.03) and lighter tails (kurtosis = 0.69) (Figure 3a).

The mean values and CV of NPQ were 0.96 and 29.13% for shaded leaves, and 0.55 and 42.54% for sunlit leaves. The NPQ of shaded leaves ranged from 0.49 to 1.54, whilst that of sunlit leaves ranged from 0.24 to 1.22. The density distribution of NPQ showed a skewness and kurtosis of 0.29 and −1.06 for shaded leaves and 1.43 and 1.01 for sunlit leaves (Figure 3b). The qL values for shaded and sunlit leaves ranged from 0.08 to 0.51 and 0.26 to 0.74, respectively. The CV was 42.08% for shaded leaves and 18.65% for sunlit leaves. A negatively skewed distribution (skewness = −0.08) with lighter tails on the left side (kurtosis = −1.39) was observed for the qL in shaded leaves. In comparison, the distribution for sunlit leaves was positively skewed (skewness = 0.11), with a slight right tail (kurtosis = 0.22) (Figure 3c).

ΦP varied within the range of 0.09 to 0.53, with a CV of 34.12% for shaded leaves. In sunlit leaves, ΦP ranged from 0.38 to 0.66, and its CV was 9.89%. There were negatively skewed distributions for ΦP in shaded (skewness = −0.13) and sunlit (skewness = −0.51) leaves, with a left tail (kurtosis = −1.19) and a right tail (kurtosis = 0.42), respectively (Figure 3d). ΦN of shaded leaves had a skewness of −0.03 and kurtosis of −0.99, whilst ΦF had a skewness of 0.32 and kurtosis of −0.18. The skewness and kurtosis values in sunlit leaves were 0.84 and 0.21 for ΦN (Figure 3e), and 0.08 and 0.23 for ΦF (Figure 3f), respectively. Furthermore, the ΦN of shaded and sunlit leaves exhibited ranges from 0.16 to 0.55 and 0.09 to 0.30, with mean values of 0.33 and 0.16 and CVs of 26.54% and 31.23%, respectively. The ΦF had mean values of 0.019 and 0.016, with CVs of 15.55% and 13.35% for shaded and sunlit leaves, respectively.

### 3.2. Performance of Published Spectral Indices

The efficacy of distinctive published spectral indices in estimating ChlF parameters was evaluated for shaded and sunlit leaves (Table 4). Among the various reported spectral indices, PRI demonstrated the most favorable performance for tracking PSII_max_ in both shaded and sunlit leaves. However, its R^2^ value was very low (R^2^ = 0.052 for shaded leaves and 0.045 for sunlit leaves), and the predictive ability of this index was not particularly convincing (*p* > 0.05). For NPQ, RSI and RGI had the highest R^2^ in shaded (R^2^ = 0.071, *p* < 0.05) and sunlit leaves (R^2^ = 0.070, *p* < 0.05), respectively. PRI achieved an R^2^ of 0.103 for tracking the qL in shaded leaves (*p* < 0.01). For sunlit leaves, none of the reported indices performed well for estimating the qL, with the best-performing index being PSRI, which had an R^2^ of only 0.036 (*p* > 0.05). For the ΦP of shaded and sunlit leaves, the best-performing indices were PRI and EVI, with R^2^ values of 0.118 (*p* < 0.01) and 0.050 (*p* > 0.05), respectively. RGI was the best index for evaluating ΦN in sunlit leaves (R^2^ = 0.066, *p* < 0.05). For shaded leaves, ARI2 was the best-performing index for ΦN (R^2^ = 0.114, *p* < 0.01). PRI was found to be effective for the tracking of ΦF in shaded leaves (R^2^ = 0.113 *p* < 0.01), while the YCAR method demonstrated the greatest performance, although not to a statistically significant degree (R^2^ = 0.034, *p* > 0.05), in sunlit leaves.

### 3.3. Performance of Spectral Indices Derived from Different FOD Spectra and Spectral Transformations

**Figure 4:**
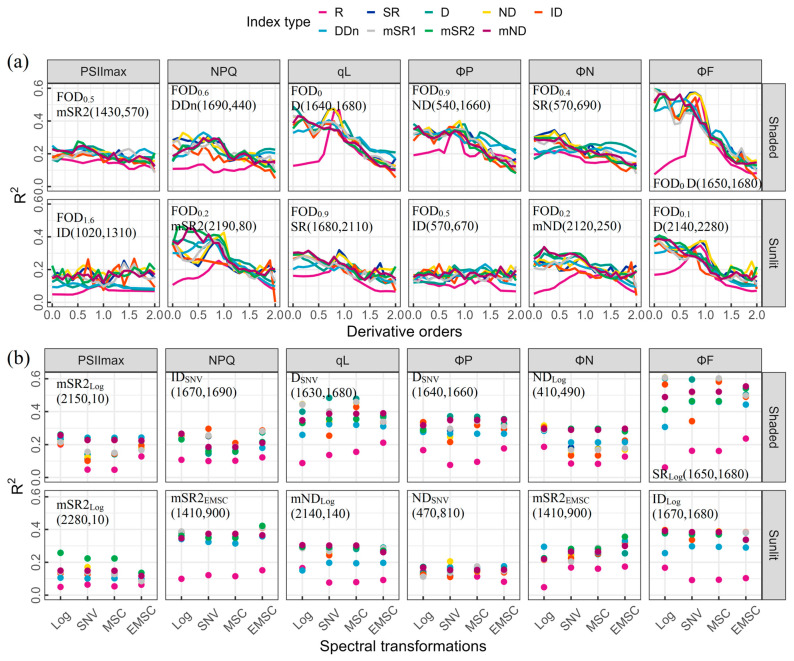
Performance of different index types in estimating ChlF parameters of shaded and sunlit leaves calculated from various (**a**) FOD spectra and (**b**) spectral transformations. Color-coded by index type.

Spectral indices of different index types, derived from different FOD spectra, were compared (Figure 4a). For shaded leaves and sunlit leaves, the optimal spectral indices developed were all based on low-order FOD spectra (<1), except for the PSII_max_ of sunlit leaves. The D type index calculated from 0-order spectra based on wavelengths of 1640, 1650, and 1680 nm effectively captured the qL (R^2^ = 0.49) and ΦF (R^2^ = 0.60) of shaded leaves. The PSII_max_, NPQ, ΦP, and ΦN of shaded leaves were accurately estimated using the mSR2 (1430, 570), DDn (1690, 440), ND (540, 1660), and SR (570, 690) indices, respectively (R^2^ = 0.28, R^2^ = 0.33, R^2^ = 0.40, and R^2^ = 0.34, respectively), derived from 0.5-, 0.6-, 0.9-, and 0.4-order spectra, respectively. The best index type for tracking the PSII_max_ (R^2^ = 0.27) and ΦP (R^2^ = 0.23) of sunlit leaves was the ID type, based on 1.6- and 0.5-order spectra, respectively. The wavelengths were 1020 and 1310 nm for PSII_max_ and 570 and 670 nm for ΦP. For other ChlF parameters of sunlit leaves, the mSR2 (2190, 80) for NPQ (R^2^ = 0.46), SR (1680, 2110) for qL (R^2^ = 0.32), mND (2120, 250) for ΦN (R^2^ = 0.34), and D (2140, 2280) for ΦF (R^2^ = 0.41) were defined, based on 0.2-, 0.9-, 0.2-, and 0.1-order spectra, respectively. 

The efficacy of various spectral indices calculated from Log, SNV, MSC, and EMSC transformations in estimating ChlF parameters for shaded and sunlit leaves was compared (Figure 4b). Based on Log transformations, mSR2 (2150, 10), ND (410, 490) and SR (1650, 1680) showed the best performance in estimating the PSII_max_ (R^2^ = 0.26), ΦN (R^2^ = 0.32), and ΦF (R^2^ = 0.61) of shaded leaves, respectively. Additionally, the ID (1670, 1690) and D ((1630, 1680) and (1640, 1660)) index types, based on SNV transformations, effectively tracked the NPQ (R^2^ = 0.30), qL (R^2^ = 0.48), and ΦP (R^2^ = 0.37) of shaded leaves, respectively. For sunlit leaves, mSR2 (2280, 10), mND (2140, 140), and ID (1670, 1680) after Log transformations, proved to be the best indices to evaluate PSII_max_ (R^2^ = 0.26), qL (R^2^ = 0.30), and ΦF (R^2^ = 0.40), respectively. Additionally, mSR2 (1410, 900) based on an EMSC transformation showed the best performance in tracking NPQ (R^2^ = 0.42) and ΦN (R^2^ = 0.36). As for ΦP, the ND (470, 810) calculated from an SNV transformation emerged as the best index, with an R^2^ of 0.21.

### 3.4. Considering the Effect of Specific Species on ChlF Parameters’ Estimation

**Figure 5:**
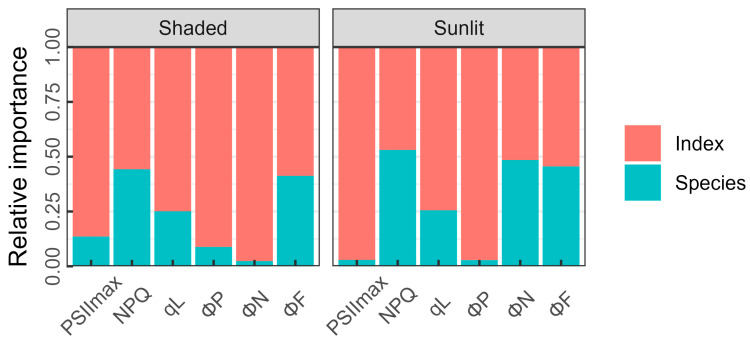
The relative importance of developed indices and species specificity for the ChlF parameters of shaded and sunlit leaves.

The relative importance of the identified optimal spectral indices and species specificity for the estimation of the ChlF parameters of shaded and sunlit leaves was evaluated (Figure 5). The relative importance of the developed spectral indices was 0.86 and 0.97 for PSII_max_ and 0.91 and 0.97 for ΦP in shaded and sunlit leaves, respectively, whilst species contributed minimally. Species-specific effects had significant impacts on the estimation of the NPQ, qL, and ΦF in shaded leaves (0.44, 0.25, 0.41) and sunlit leaves (0.53, 0.26, 0.46). For ΦN, the relative importance of species was low in shaded leaves (0.02), but higher in sunlit leaves (0.48). 

**Figure 6:**
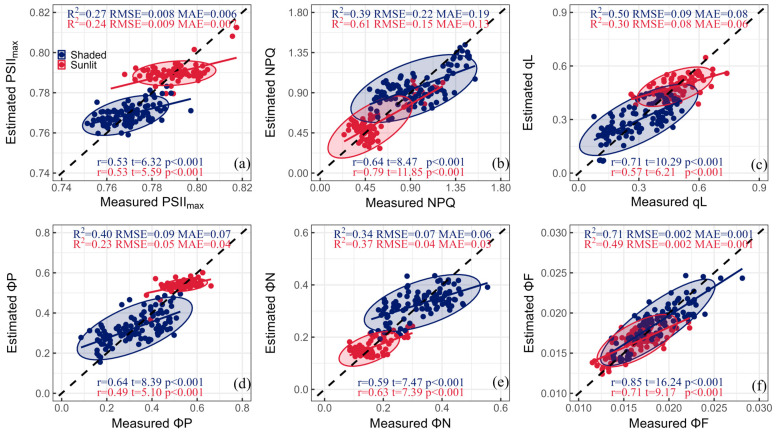
Measurements and predictions of (**a**) PSII_max_, (**b**) NPQ, (**c**) qL, (**d**) ΦP, (**e**) ΦN, and (**f**) ΦF based on multiple linear regression combining optimal spectral indices and the species-specific factor for shaded (blue) and sunlit (red) leaves. Model performance metrics, including the coefficient of determination (R^2^), Pearson coefficient (r), root mean square error (RMSE), and mean absolute error (MAE), are provided. The t-value measures the strength of the relationship, while the *p*-value indicates the significance of this correlation.

Figure 6 shows the results of a multiple linear regression using optimal spectral indices and species for the estimation of ChlF parameters in shaded and sunlit leaves. The combination of optimal spectral indices and species-specific effects could accurately track the NPQ of sunlit leaves and the qL and ΦF of shaded leaves (Figure 6b,c,f), with high R^2^ values of 0.61, 0.50, and 0.71, respectively. At the same time, the r was 0.79, 0.71, and 0.85 for the parameters mentioned above, indicating strong relationships between the predicted and observed values. Due to the low relative contribution of species-specific effects to PSII_max_ and ΦP (Figure 5), no improvement in R^2^ values was observed for shaded (R^2^ = 0.27 for PSII_max_ and 0.40 for ΦP) and sunlit leaves (R^2^ = 0.24 for PSII_max_ and 0.23 for ΦP) (Figure 6a,d). For ΦN, the integration of the developed spectral index and specific species explained 34% and 37% of the total variance in shaded leaves and sunlit leaves, respectively (Figure 6e).

## 4. Discussion

### 4.1. FOD Spectra and Spectral Transformations Optimize the Performance of Spectral Indices in Estimating ChlF Parameters

This study demonstrated that the performance of published spectral indices in tracking ChlF parameters was limited (Table 4). Even the best-performing PRI for estimating the ΦP of shaded leaves had an R^2^ of only 0.118. Other published spectral indices had even lower R^2^ values, with some being statistically unreliable (*p* > 0.05). Additionally, differences in ChlF parameters between shaded and sunlit leaves were observed (Table 3 and Figure 3). However, the published empirical spectral indices were not sensitive enough to estimate ChlF parameters in sunlit and shaded leaves (Table 4). A previous study on *Mangifera indica* L. demonstrated that the reliability of the reported spectral indices in estimating ChlF parameters was low for shaded and sunlit leaves [24]. In addition, an experiment in temperate deciduous forests evaluated the potential of 30 reported spectral indices to track ChlF parameters. However, no well-known connection between PRI and NPQ was observed, and no specific index was proposed for the estimation of ChlF parameters [21]. Although a high correlation between RSI and PSII_max_ and ΦP was observed in *Suaeda salsa* L. under salinity stress [17], this may be because the empirical spectral index is more sensitive to ChlF parameters under stress [33]. Physiological traits under stress can optimize the linear relationship between the empirical index and the photochemical process [34]. Therefore, the ChlF parameter of the empirical spectral index may be useful under stress conditions, but it is necessary to be cautious about its applicability under other conditions, especially for data containing different leaf groups and species.

The utilization of FOD spectra and various spectral transformations to calculate different types of spectral indices for estimating leaf biochemical and photosynthetic capacity parameters is superior [26,29,31]. However, there are few reports on tracking ChlF parameters using a dense interval of FOD spectra, and existing studies are limited to integer-order derivative spectra. For example, a previous study on grapes found that the spectral index calculated using the first derivative at 735 and 544 nm could explain 68% of the variation in PSII_max_. Meanwhile, the first derivative at 676 nm and the original spectral reflectance at 571 nm could explain 63% of ΦP [35]. Zheng et al. [36] estimated the NPQ of *Suaeda salsa* L. under water and salt conditions; the Pearson correlation coefficient reached 0.745 when utilizing wavelengths of 1480 and 954nm based on first-order derivative spectra. In this study, the accuracy of ChlF parameters’ estimation of different leaf groups (shaded and sunlit) was improved through the use of FOD spectra and different spectral transformations (Figure 4). Our study further demonstrated that, with increasing the order of FOD spectra at a low level (<1), better spectral indices could be constructed to capture the ChlF parameters of shaded and sunlit leaves (except for the PSII_max_ of sunlit leaves). However, spectral indices constructed based on higher-order FOD spectra (>1) performed poorly (Figure 4a). Similar findings were observed in the estimation of LMA, the maximum carboxylation rate, and the maximum electron transfer rate [26,37]. Their study emphasized the superiority of low-order FOD spectra. This may be because low-order FOD spectra amplify the spectral information used to estimate ChlF parameters, while high-order FOD spectra are sensitive to noise and thus obscure useful spectral information [38]. In addition, the spectral indices constructed based on SNV, Log, and EMSC have higher accuracy than FOD spectra for the ΦF of shaded leaves and ΦN of sunlit leaves, respectively (Figure 4b). Wen et al. [39] highlighted the correlation between spectral reflectance characteristics and ChlF parameters by performing SNV and MSC transformations on the leaf spectra of rice. Similarly, it has been demonstrated that log-transformed and EMSC-converted spectra are advantageous in capturing PSII_max_ and ΦF [24]. Therefore, low-order FOD spectra and appropriate spectral transformations are beneficial approaches to retrieving ChlF parameters.

### 4.2. Effect of Species Specificity on the Estimation of ChlF Parameters

This study further improved the simulation performance of ChlF parameters for different leaf groups by developing an optimal spectral index and accounting for species-specific effects (Figure 6). For the NPQ of sunlit leaves and the qL and ΦF of shaded leaves, these R^2^ values reached 0.61, 0.50, and 0.71, respectively (Figure 6b,c,f). This reflects the variations in light tolerance between shaded and sunlit leaves of different species. A previous experiment involving various woody and herbaceous species identified significant differences in NPQ between species when leaves were grown under varying irradiances [15]. For instance, *Tilia cordata* Mill. promptly adjusted its NPQ to mitigate the photodamage induced by high irradiance. Furthermore, disparities in photochemical capacity among species can influence their light tolerance, as a higher photochemical capacity offers crucial protection against damage from high light intensity, a characteristic often reflected in the qL [40,41]. ΦF characterizes the proportion of fluorescent quantum yields to absorbed radiation and has been shown to vary between species [42]. To summarize, our research has enhanced the predictive capacity of these parameters by accounting for species-specific effects.

After accounting for species variables, the estimation of PSII_max_ and ΦP showed limited improvement (Figure 6a,d). The range of PSII_max_ for mature healthy leaves is generally between 0.75 and 0.85. In this study, the variation in PSII_max_ was minimal for shaded and sunlit leaves, with a CV of 1.16% and 1.21%, respectively. Additionally, PSII_max_ and ΦP are known to be closely related to leaf traits and pigment content [43]. Recent research has indicated that light intensity and light composition play a pivotal role in capturing the changes in ΦP [22]. The simulation accuracy of PSII_max_ and ΦP could be improved by considering additional leaf biochemical and environmental factors. Consequently, the inclusion of species variables had a limited effect on improving the simulation accuracy of PSII_max_ and ΦP.

### 4.3. Uncertainty and Prospects

We need to be cautious about the sensitive bands used to estimate ChlF parameters. In this study, the bands with the best indices for estimating ChlF parameters were mainly concentrated in the visible light (400–680), red edge (681–780), near-infrared (781–1400), and part of the short-wave infrared regions (1401–2400) (Figure 4). These regions are intimately associated with leaf compounds, pigment content, structure, and water status [44,45,46]. Comprehensive leaf traits serve to determine ChlF parameters and are reflected in the reflectance spectra [47]. For PSII_max_, the optimal bands for shaded and sunlit leaves were located in the near-infrared region. This indicates that the spectral index derived from this region is suitable for estimating PSII_max_ due to the strong absorption of red light by chlorophyll and the reabsorption of fluorescence caused by the multiple scattering of light within leaves [48]. The small differences in the bands determined for shaded and sunlit leaves may be due to the varying sensitivity of PSII_max_ to leaf structure and water status parameters in different leaf groups. Similarly, the bands used for predicting ΦP emphasize the importance of leaf pigment content and water status, with sunlit leaves being more sensitive to leaf pigment content. Consistent with our findings, previous studies have suggested that spectral indices constructed based on the red edge and short-wave infrared regions can effectively capture changes in PSII_max_ and ΦP [36]. The high sensitivity of NPQ to the short-wave infrared band indicates that the leaf water status and biochemical components, such as proteins, significantly influence the estimation of NPQ. Similar wavelength selections have been widely reported in the literature [49,50,51]. For shaded leaves, the wavelength information utilized for NPQ prediction encompasses near-infrared regions, thereby emphasizing the distinctive impact of leaf structure.

This study demonstrated that spectral indices constructed based on low-order FOD spectra or appropriate spectral transformation methods, while considering species-specific effects, can effectively track a series of ChlF parameters. However, their predictions of PSII_max_ and ΦP were poor. This suggests that spectral information based on a limited number of wavelengths (1–3) may not adequately capture the dynamics of ChlF parameters, especially in multi-species datasets. Therefore, using a principal component analysis (PCA) to reduce the dimensionality of spectral data and enhance the interpretability of spectral features is a reliable technique [52]. A study by Falcioni et al. [53] used a PCA to combine fifteen components in *Nicotiana tabacum* L. spectra to simulate ChlF parameters, demonstrating its feasibility. Additionally, methods such as partial least squares regression, machine learning, and deep learning, which rely on feature selection, are worth considering [54,55,56]. Future studies should also strengthen our understanding of the mechanisms at work between leaf traits, spectral reflectance signals, and ChlF parameters. This will help in selecting spectral bands that contain the richest and most effective information to quickly and accurately track ChlF parameters, providing a basis and insights for the remote sensing monitoring of ChlF parameters.

## 5. Conclusions

As a promising spectral preprocessing method, FOD offers a novel approach to monitoring ChlF parameters based on spectral reflectance information. The performance of spectral indices developed based on low-order FOD in tracking ChlF parameters has demonstrated an improvement in comparison to empirical spectral indices. Moreover, the accuracy of the simulations was further enhanced by incorporating an optimal spectral index and species-specific effects, particularly for NPQ, qL, and ΦF. Nevertheless, the inherent complexity of ChlF parameters presents challenges when estimating them using spectral indices alone. Future studies should consider the potential benefits of leveraging a combination of multiple sensitive bands to enhance their assessment capabilities.

## Figures and Tables

**Table 1:** Calculation of ChlF parameters. KP, KN, KF, and KD denote the rate of photochemistry, energy-dependent heat dissipation, fluorescence, and constitutive heat dissipation, respectively.

Parameter	Calculation	Reference
PSII_max_	$\frac{F_{m}-F_{0}}{F_{m}}$	Kitajima and Butler [7]
NPQ	$\frac{F_{m}-F_{m}^{\prime }}{F_{m}^{\prime }}$	Bilger and Björkman [8]
qL	$qL=\frac{F_{m}^{\prime }-F_{s}}{F_{m}^{\prime }-F_{0}^{\prime }}\times \frac{F_{0}^{\prime }}{F_{s}}$	Miyake, Amako, Shiraishi, and Sugimoto [6]
	$F_{0}^{\prime }=\frac{F_{0}}{{PSII}_{max}-\frac{F_{0}}{F_{m}^{\prime }}}$	Oxborough and Baker [5]
ΦP	$\Phi P=\frac{K_{P}}{\sum K}$	Butler [4]
	$K_{P}=\frac{F_{m}^{\prime }-F_{s}}{F_{s}}\times \left(K_{F}+K_{D}+K_{N}\right)$	
ΦN	$\Phi N=\frac{K_{N}}{\sum K}$	
	$K_{N}=\frac{F_{m}-F_{m}^{\prime }}{F_{m}^{\prime }}\times \left(K_{F}+K_{D}\right)$	
	$K_{D}=Max(0.03\times T+0.0773,0.87)$	
ΦF	$\Phi F=\frac{K_{F}}{\sum K}$	
	$K_{F}=0.05$	

**Table 2:** Spectral index types for the estimation of ChlFa parameters.

Index	Wavelength	Formula
given wavelength (R)	λ1	$R_{\lambda 1}$
simple ratio (SR)	λ1 and λ2	$R_{\lambda 1}/R_{\lambda 2}$
wavelength difference (D)	λ1 and λ2	$R_{\lambda 1}-R_{\lambda 2}$
normalized difference (ND)	λ1 and λ2	$\left(R_{\lambda 1}-R_{\lambda 2})/(R_{\lambda 1}+R_{\lambda 2}\right)$
inverse differences (ID)	λ1 and λ2	$\left(\frac{1}{R_{\lambda 1}})-(\frac{1}{R_{\lambda 2}}\right)$
double differences (DDn)	λ1 and Δλ	2$R_{\lambda 1}-R_{\lambda 1-\Delta \lambda }-R_{\lambda 1+\Delta \lambda }$
modified simple ratio 1 (mSR1)	λ1 and Δλ	($R_{\lambda 1-\Delta \lambda }-R_{\lambda 1})/R_{\lambda 1+\Delta \lambda }$
modified simple ratio 2 (mSR2)	λ1 and Δλ	($R_{\lambda 1-\Delta \lambda }-R_{\lambda 1})/{(R}_{\lambda 1+\Delta \lambda }-R_{\lambda 1})$
modified normalized difference (mND)	λ1 and Δλ	($R_{\lambda 1-\Delta \lambda }-R_{\lambda 1})/{(R}_{\lambda 1-\Delta \lambda }+R_{\lambda 1}-2R_{\lambda 1+\Delta \lambda })$

**Table 3:** Statistical descriptions of ChlF parameters for shaded and sunlit leaves.

Leaf Group	Parameters	Mean	Median	Minimum	Maximum	CV (%)	Skewness	Kurtosis
Shaded	PSII_max_	0.77	0.77	0.75	0.80	1.16	0.40	−0.18
	NPQ	0.96	0.91	0.49	1.54	29.13	0.29	−1.06
	qL	0.30	0.30	0.08	0.51	42.08	−0.08	−1.39
	ΦP	0.33	0.33	0.09	0.53	34.12	−0.13	−1.19
	ΦN	0.33	0.34	0.16	0.55	26.54	−0.03	−0.99
	ΦF	0.019	0.019	0.014	0.028	15.55	0.32	−0.18
Sunlit	PSII_max_	0.79	0.79	0.76	0.82	1.21	−0.03	0.69
	NPQ	0.55	0.49	0.24	1.22	42.54	1.43	1.01
	qL	0.48	0.48	0.26	0.74	18.65	0.11	0.22
	ΦP	0.54	0.54	0.38	0.66	9.89	−0.51	0.42
	ΦN	0.16	0.15	0.09	0.30	31.23	0.84	0.21
	ΦF	0.016	0.016	0.012	0.022	13.35	0.08	0.23

**Table 4:** Performance of published spectral indices in predicting ChlF parameters for shaded and sunlit leaves.

Leaf Group	Index	PSII_max_		NPQ		qL		ΦP		ΦN		ΦF	
		R^2^	RMSE	R^2^	RMSE	R^2^	RMSE	R^2^	RMSE	R^2^	RMSE	R^2^	RMSE
Shaded	ARI2	0.018	0.008	0.042	0.269	0.054	0.123	0.087	0.099	0.114	0.074	0.005	0.003
	CRI1	0.001	0.009	0.017	0.272	0.008	0.126	0.002	0.104	0.008	0.078	0.020	0.003
	CRI2	0.002	0.008	0.009	0.273	0.017	0.126	0.0046	0.104	0.001	0.078	0.025	0.003
	EVI	0.037	0.008	0.032	0.270	0.022	0.125	0.012	0.103	0.001	0.078	0.056	0.003
	OCAR	0.010	0.008	0.016	0.272	0.056	0.123	0.082	0.010	0.066	0.076	0.026	0.003
	PRI	0.052	0.008	0.003	0.275	0.103	0.120	0.118	0.098	0.040	0.077	0.113	0.003
	PSRI	0.006	0.008	0.058	0.267	0.008	0.126	0.039	0.102	0.063	0.076	0.001	0.003
	RGI	0.018	0.008	0.060	0.266	0.011	0.126	0.042	0.102	0.086	0.075	0.008	0.003
	RSI	0.002	0.008	0.071	0.265	0.003	0.127	0.004	0.104	0.044	0.077	0.027	0.003
	YCAR	0.009	0.008	0.019	0.272	0.046	0.124	0.072	0.100	0.067	0.076	0.017	0.003
Sunlit	ARI2	0.009	0.009	0.015	0.246	0.005	0.091	0.038	0.053	0.029	0.051	0.001	0.002
	CRI1	0.011	0.009	0.001	0.247	0.001	0.091	0.002	0.054	0.001	0.052	0.001	0.002
	CRI2	0.010	0.009	0.001	0.247	0.003	0.091	0.003	0.054	0.001	0.052	0.001	0.002
	EVI	0.020	0.009	0.002	0.247	0.025	0.090	0.050	0.053	0.011	0.052	0.024	0.002
	OCAR	0.001	0.009	0.031	0.244	0.027	0.090	0.003	0.054	0.018	0.052	0.031	0.002
	PRI	0.045	0.009	0.010	0.246	0.009	0.091	0.002	0.054	0.003	0.052	0.016	0.002
	PSRI	0.020	0.009	0.023	0.245	0.036	0.089	0.002	0.054	0.010	0.052	0.032	0.002
	RGI	0.039	0.009	0.070	0.239	0.011	0.090	0.020	0.053	0.066	0.050	0.018	0.002
	RSI	0.019	0.009	0.003	0.247	0.001	0.091	0.004	0.054	0.001	0.052	0.002	0.002
	YCAR	0.013	0.009	0.044	0.242	0.032	0.090	0.004	0.054	0.029	0.051	0.034	0.002

## Data Availability

Dataset available on request from the authors.

## Supplementary Materials

The following supporting information can be downloaded at https://www.mdpi.com/article/10.3390/plants13141923/s1, Table S1: Published spectral indices for the estimation of ChlFa parameters from previous studies.
