# Supplementary material for: Hyperspectral Indices Developed from Fractional-Order Derivative Spectra Improved Estimation of Leaf Chlorophyll Fluorescence Parameters
Source: Plants (Basel). 2024 Jul 12;13(14):1923. doi: 10.3390/plants13141923 (PMC11281006; doi:10.3390/plants13141923)
Supplement: Supplementary file 1 [file plants-13-01923-s001.zip › plants-3083006-supplementary.pdf]

Supplementary materials

Table S1. Published spectral indices for the estimation of ChlFa parameters in other studies. R represents the reflectance.

Index name	Formula	Reference
ARI2	$R_{800} * (1/R_{550} - 1/R_{700})$	Stratoulias, et al. [19]
CRI1	$1/R_{510} - 1/R_{550}$	Stratoulias, et al. [19]
CRI2	$1/R_{510} - 1/R_{700}$	Stratoulias, et al. [19]
EVI	$2.5 * (R_{780} - R_{675})/(R_{782} + 6 * R_{675} - 7.5 * R_{445} + 1)$	Stratoulias, et al. [19]
OCAR	R_{630}/R_{680}	Zhang, et al. [17]
PRI	$(R_{531} - R_{570})/(R_{531} + R_{570})$	Stratoulias, et al. [19]
PSRI	$(R_{680} - R_{500})/R_{750}$	Stratoulias, et al. [19]
RGI	R_{690}/R_{550}	Stratoulias, et al. [19]
RSI	R_{680}/R_{935}	Zhang, et al. [17]
YCAR	R_{600}/R_{680}	Zhang, et al. [17]
